# Fundamentals of Presbyopia: visual processing and binocularity in its transformation

**DOI:** 10.1186/s40662-018-0095-0

**Published:** 2018-01-25

**Authors:** Olga I. Rozanova, Andrey G. Shchuko, Tatyana S. Mischenko

**Affiliations:** 1 Irkutsk branch of S. Fyodorov Eye Microsurgery Federal State Institution, Irkutsk, Russian Federation; 2 Irkutsk State Medical University, Irkutsk, Russian Federation

**Keywords:** Presbyopia, Binocularity, Visual processing, Visual reception

## Abstract

**Background:**

The accommodation has considerable interactions with the pupil response, vergence response and binocularity. The transformation of visual reception processing and the changes of the binocular cooperation during the presbyopia development are still poorly studied. So, the regularities of visual system violation in the presbyopia formation need to be characterized. This study aims to reveal the transformation of visual reception processing and to determine the role of disturbances in binocular interactions in presbyopia formation.

**Methods:**

This study included 60 people with emmetropic refraction, uncorrected distance visual acuity 1.0 or higher (decimal scale), normal color perception, without concomitant ophthalmopathology. The first group consisted of 30 people (from 18 to 27 years old) without presbyopia, the second cohort comprised 30 patients (from 45 to 55 years old) with presbyopia. The eyeball anatomy and optics were evaluated using ultrasound biomicroscopy, aberrometry, and pupillometry. The functional state of the visual system was investigated under monocular and binocular conditions. The registration of the disparate fusional reflex limits was performed by the original technic using a diploptic device which facilitated investigation of the binocular interaction under natural conditions without the accommodation response, but with the different vergence load. The disparate fusional reflex was analyzed using the proximal and distal fusion borders, and the convergence and divergence fusion borders. The calculation of the area of binocularity field was performed in cm^2^.

**Results:**

The presbyopia formation is characterized by a change in an intraocular anatomy, optics, visual processing, and binocularity. The processes of binocular interaction inhibition make a significant contribution to the misalignment of the visual perception. The modification of the proximal, distal and convergence fusion borders was determined. It was revealed that 87% of the presbyopic patients had binocularity shortage, whereas the reduction of binocularity field area in extreme grade was seen in 6% of cases.

**Conclusions:**

The presbyopia formation is accompanied by a significant reorganization of the visual system activity and by the creation of the new visual processing interactions. These data may be useful in presbyopia surgery.

## Background

According to the International Classification of Diseases (ICD-10, version: 2010) presbyopia belongs to the class of refraction and accommodation disturbance and is slow, age-related and irreversible accommodation decline. Currently, over 1.7 billion people in the world are afflicted with presbyopia [1]. The problem appears in people at the age of 40 – 45 years, which is the period of maximal professional and creative activity involving analyses of a significant volume of visual information [2–4].

The problem of presbyopia correction is an actual task, but in spite of the active introduction of advanced methods, the question about adequate presbyopia correction remains open and unsettled [5]. For the compensation of accommodation deficit, different variants of a multifocal optical system creation have been described [1, 5–12].

But the creation of a multifocal monocular optical system or anisometropic binocular optical system is closely associated with the adaptation processes, which are very complicated in some patients. The multifocal monocular optical system does not have any physiological analogs [13–15]. Regardless of the multifocal IOL model, there is always a proportion of patients who complain of the visual dysphotopsia as blurring, misting, “holographic” view, 3D - view. In 5% of cases, this dysphotopsia syndrome becomes rigid and is an indication for IOL explantation [16]. The reasons for the visual dysphotopsia are not clear enough. The explantation of multifocal intraocular lens is debated as an “army method of refractive surgery” [16] and is about 3 to 10% according to the data by different authors [16–20].

Most of the presbyopia theories consider the intraocular changes purely. The presbyopia is mainly viewed as an accommodation decrease determined by the reduction of lenticular elasticity and changing lens suspension apparatus [21–24]. At the same time, the accommodative response is a part of the near synkinetic reflex. The accommodation has significant interactions with the pupil response, vergence response, and binocularity.

However, based on the theory of functional systems, the loss of useful component in the body’s activity is accompanied by the measures to compensate or to adapt for it [25–27]. Therefore, the reduction of the accommodative response that underlies the presbyopia development must be inevitably accompanied by the imbalance among the components of the near synkinetic reflex. How does the accommodation decrease influence the ocular motor forces which are responsible for the stable constant visual image? This problem has not been solved yet.

The changes of the binocular cooperation during the presbyopia development are still poorly studied. According to our previous studies, there is some deficit of binocular cooperation in age-related accommodation loss [28]. Besides, the aging-induced accommodation decrease is strongly linked with other physiological aging processes and the degradational changes of sensory neurons. The normal aging is accompanied by the visual acuity decrease, contrast sensibility decrease, color perception changing, stereoacuity decrease [29–32]. The age-related reduction of stereovision is marginally correlated with fusional ability decrease [33]. But what remains unclear is the role of binocular disorders among mechanisms underlying presbyopia pathogenesis. Therefore, comprehending the binocular interaction changes in people with presbyopia is a critical issue, and the regularities of visual system violation in the presbyopia formation need to be characterized.

The purpose of this study is to examine the transformation of visual reception processing and to determine the role of binocular interactions disturbance in presbyopia formation.

## Methods

### Subjects

The study adhered to the tenets of Helsinki Declaration and was approved by the Institution Research and Ethics Committee (protocol number 8/13 from 25/11/2013). All patients were adequately informed and signed a consent form. Of the 60 people examined, the first group consisted of 30 people (from 18 to 27 years old) without presbyopia, and the second group comprised 30 patients (from 45 to 55 years old) with presbyopia.

The criteria for inclusion in this study comprise the presence of emmetropic refraction (i.e., the spherical equivalent of cycloplegic measurements from +0.25 D to −0.25 D), uncorrected distance visual acuity of each eye 1.0 (decimal scale) or higher, normal color perception, ophthalmopathology absence. All patients of the second group complained of the insufficient near vision, and they made use of the glasses.

Considering that binocularity correlates the heterophoria value and interpupillary distance [34, 35], the exclusion criteria comprised a heterophoria degree greater than five prism diopter, the pupillary distance less than 62 mm and greater than 64 mm.

#### Measurements

All patients had a full ophthalmological examination including the evaluation of 80 parameters of eye anatomy, visual processing, and binocularity.

### Assessment of the eye anatomy and the physiological optics

The refractive error was the average spherical equivalent of five cycloplegic measurements taken with an autorefractor/keratometer (KR8800, Topcon, Japan). All cycloplegic measurements were made 25 min after the administration of 1% tropicamide (2 drops during 5 min interval twice).

The other investigations were performed 7-10 days later.

The amplitude of accommodation (AA) was measured using a minus lens method. The subjects were asked to fixate N8 target at a distance of 40 cm. Then minus lenses were introduced in 0.25 D steps until the patient reported the first sustained blur that could not be cleared by the further conscious effort. This procedure was done for each eye first monocularly and then binocularly. The total AA was estimated as the endpoint minus lens which was possible to see the target at 40 cm under binocular conditions. The AA measurement in people with presbyopia was done using the near addition lens.

The axial length, lens thickness, anterior chamber (AC) measurements were made with the help of an ultrasound biometer (AL-3000, Tomey, Japan). An average of three measurements for each parameter was used. Ultrasound biomicroscopy (UBM) measurements were made using HiScan 2000 (Optikon, Italy), and UBM was done in the supine position as described by C. Pavlin and F. Foster [36]. Images from the iris root to the pars plicata zone were obtained in 12 o’clock direction. The ciliary body thickness in cross section and the length of anterior portion Zinn’s ligament were examined. The ocular wavefront aberration across a 3 mm zone in the pupil and the pupil diameter (under photopic and mesopic conditions) were obtained using the principle of automated retinoscopy (OPD-Scan, NIDEK, Japan). The anterior chamber volume, iris and lens configuration, corneal aberrations, lens light transmission, were fixed using Pentacam (Oculus Optikgeräte GmbH, Germany).

### Assessment of visual processing under monocular conditions

The distance visual acuity and the near visual acuity were measured with logical geometric scale Bailey-Lovie (logMAR). Other examinations included evaluation of the contrast sensitivity at spatial frequencies 3, 6, 12, 18 cycles per degree (CSV-10000E, VectorVision, USA), the threshold of light sensitivity (EP3000, Tomey, Japan), the flicker fusion threshold, the amplitude and implicit time of maximal electric retinal response, and the amplitude and implicit time of visual evoked potentials (EP1000, Tomey, Japan).

### Binocularity assessment

For this purpose, the data reflecting the different levels of binocular interaction were systematized. These include the near physiological diplopia, the stereovision test Lang I&II, and the binocularity field (spatial limits of disparate fusional reflex).

To induce the physiological diplopia, each patient was asked to look into the far distance (5 m). Then a near-lying target (in the distance 10 cm, i.e., not in the horopter curve) was presented. The presence (yes/no) of double virtual objects was fixed.

**Fig. 1:**
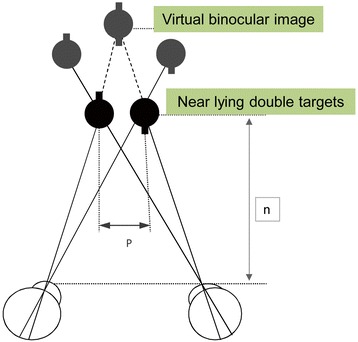
The perception of virtual binocular visual image

In the case of physiological diplopia, the registration of the disparate fusional reflex limits was performed. For this, a diploptic device (AVIS 01, Krasnogvardeec, Russia) was used, which facilitated investigation of the binocular interaction under natural conditions without the accommodation response, but with the different vergence load [37–41]. The first point of the measurement is 40 cm. Then the double targets were moved inwards until the virtual stereo image was observed (Fig. 1). It was a point of binocularity without vergence load.

**Fig. 2:**
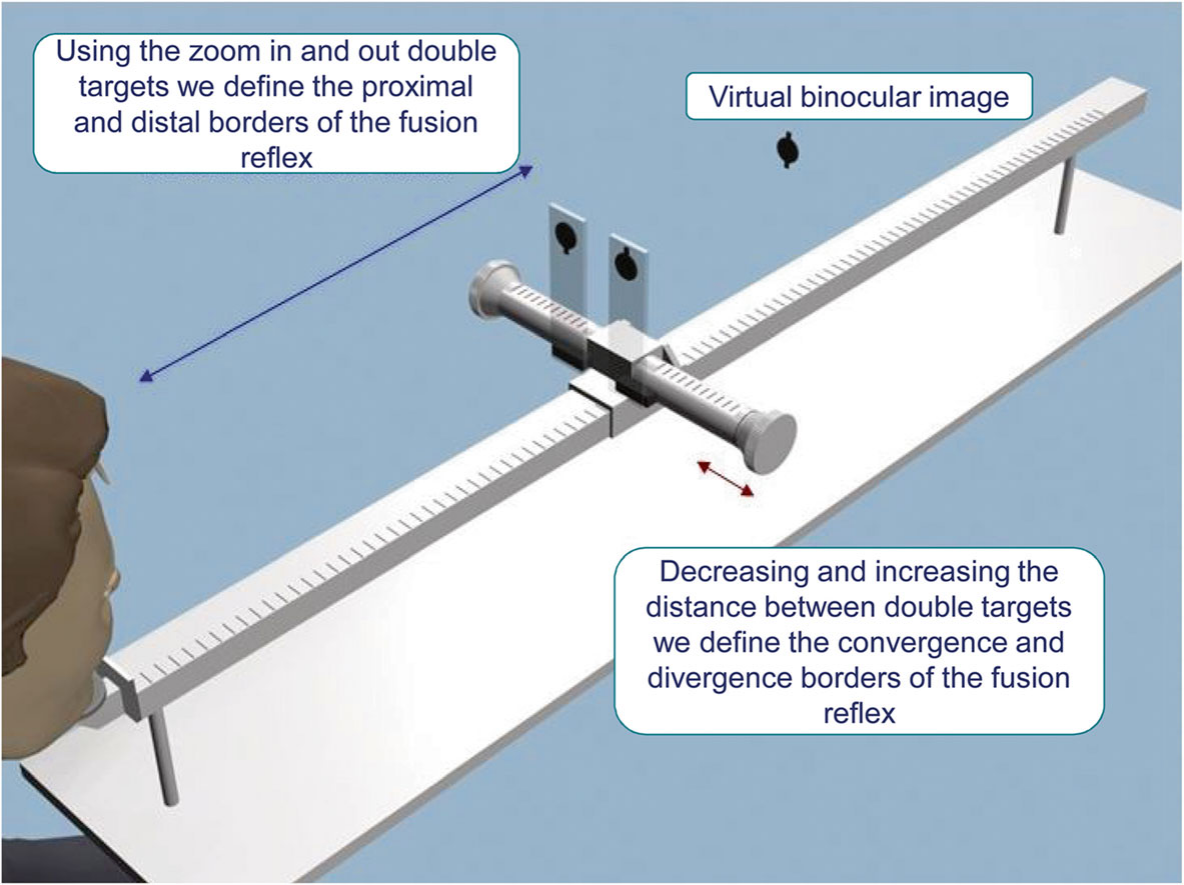
Method of Assessing the Disparate Fusion Reflex Borders

The change in the distance between the centers of the double near lying tests (p) and the distance from the eyes to the targets (n) while maintaining of the virtual binocular image perception makes it possible to define the fusional reflex limits in space. Then the targets were moved increasingly inward and outward to force the vergence response (Fig. 2). There were some points of maximum convergence and divergence. The patient reported all the visual images which were recorded.

**Fig. 3:**
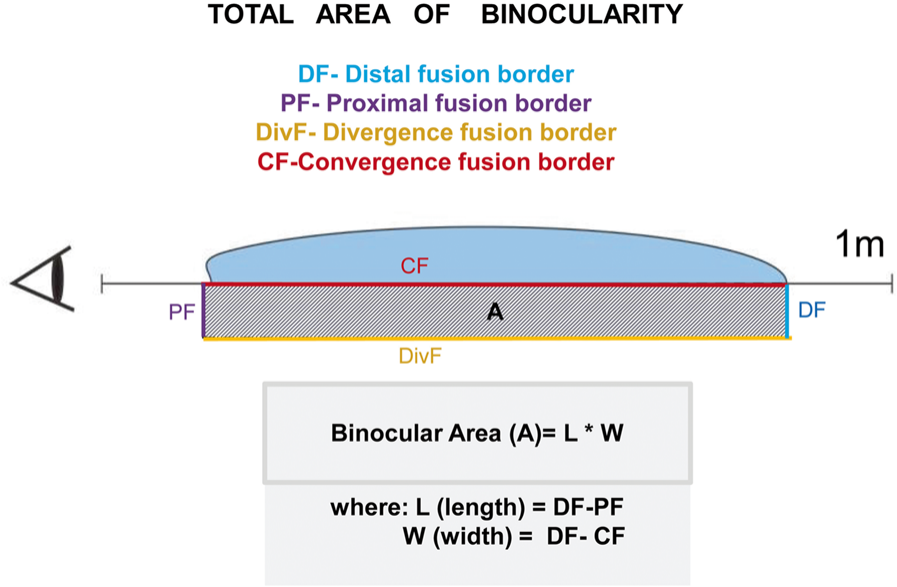
Calculation of the Binocularity Field Area

The disparate fusional reflex was analyzed using the following parameters:

1. The proximal fusion border (PF) and distal fusion border (DF) were determined while the double targets were approaching and moving away (Fig. 2). The difference between these parameters corresponds to the length of the binocularity field.
2. The convergence fusion border (CF) and the divergence fusion border (DivF) were determined with the help of the decrease and the increase of the distance between the double targets (the point of the measurement is 40 cm from the eyes). The difference between these parameters corresponds to the width of the binocularity field.
3. Finally, we performed the calculation of the binocularity field area (A) in cm^2^ (Fig. 3).

### Statistical analysis

All data were analyzed with a spreadsheet application (Statistica ver. 8.0; StatSoft Inc., USA). The data were represented as the mean value ± standard deviation (Mean ± SD)*.* The Shapiro-Wilk test was used for assessing of normality distribution. The statistical differences in measured values were analyzed using a t-test. The critical level of significance (p) upon the examination of statistical hypotheses was 0.05. The comparison analysis, the Pearson correlation analysis, and the logistic regression models were done. The Pearson correlation coefficient with absolute values equal to or greater than 0.7 with *p* < 0.001 was accepted as the close relation. The discriminant function analysis (DFA) was used for the selection of the analytes that maximally discriminated the studied groups. The DFA was built in a step-wise manner after direct standardization. The final discriminatory power of each analyte was characterized by a partial Wilk’s Lambda coefficient; 1.0 (no discriminatory power) to 0.0 (perfect discriminatory power). The Mahalanobis distance D^2^ between centroid values for each group was measured.

## Results

The baseline features of the study population were summarized in Table 1. The mean patient age was 22.3 ± 3.2 years in the first group, and 52.4 ± 2.2 years in the second group. The groups did not differ in gender, spherical refraction equivalent, eye globe axial length, oculomotor status. In the first (control) group, the mean accommodation amplitude was 6.93 ± 1.12 D (minimum 5.75 D, maximum 10.0 D). All patients with presbyopia had a decreased accommodation: the mean accommodation amplitude was 1.99 ± 0.89 D (minimum 0.5 D, maximum 4.0 D).

**Table 1:** Studied Groups Descriptive Statistics (M ± SD)

Characteristics	Control	Presbyopia	*P*-value
Age, years	22.3 ± 3.2	52.4 ± 2.2	0.0001
Female: Male	15:15	15:15	–
Mean spherical equivalent of refraction, D	0.2 ± 0.1	0.2 ± 0.2	1.00
Axial length, mm	23.5 ± 0.5	23.5 ± 0.4	1.00
Keratometry, ax 90°, D	43.2 ± 1.3	43.3 ± 1.1	0.61
Keratometry, ax 180°, D	43.0 ± 1.1	43.1 ± 1.1	0.62
Amplitude of accommodation, D	6.93 ± 1.12	1.99 ± 0.89	0.0001
Mean prism equivalent of distance heterophoria, PD	−1.2 ± 0.2	−1.1 ± 0.3	0.88

*D* = diopter; *PD* = prism diopter

### Intraocular anatomy and optics

The presbyopia formation is characterized by a change in an intraocular anatomy. Significant differences were detected in the anterior-posterior size of the lens – from 3.73 ± 0.23 to 4.41 ± 0.21 (*p* = 0.0001). The optical density (light transmission coefficient) of the lens increased in the nuclear area from 15.5 ± 1.2 to 26.6 ± 3.4% (p = 0.0001), and in the cortical layers – from 9.1 ± 0.9 to 10.8 ± 1.3% (*p* = 0.03). The change of the intraocular anatomy was expressed as: the decrease of the ciliary body thickness (in the inner top projection) from 0.82 ± 0.10 to 0.63 ± 0.11 mm (*p* = 0.001), the increase of the distance between the trabecula and the ciliary processes from 0.79 ± 0.10 to 1.02 ± 0.11 mm (p = 0.001), and the shortage of the front portion Zinn ligament length from 1.23 ± 0.31 to 1.04 ± 0.26 mm (*p* = 0.002).

The variation of both static and dynamic components of the optical physiological system was established in the eyes of presbyopic patients. This was evidenced by a significant increase of the total root mean square wavefront errors (from 0.13 ± 0.04 to 0.17 ± 0.05 μm in pupil diameter 3 mm, *p* = 0.0001) and the corneal root mean square wavefront aberrations. In patients with presbyopia, an increase in the Zernike coefficients of the corneal spherical aberration Z_4_^0^ from 0.17 ± 0.05 to 0.23 ± 0.06 μm (*p* = 0.0001) was observed. Also, a significant decrease of the pupil excursion was found. The photopic pupil diameter decreased from 3.81 ± 0.76 to 3.35 ± 0.78 mm (p = 0.0001) whereas the mesopic pupil diameter decreased from 6.47 ± 0.56 to 5.50 ± 0.94 mm (p = 0.0001).

### Monocular visual characteristics

In the next step, a comprehensive study of the sensory activity of the visual system was carried out. The patients with presbyopia had a decrease not only in accommodation amplitude and uncorrected near visual acuity but also in the most of the visual reception parameters (Table 2).

**Table 2:** Comparisons of visual characteristics in studied groups (M ± SD)

Characteristics	Control	Presbyopia	*P*-value
UDVA, logMAR	−0.071 ± 0.045	−0.003 ± 0.026	0.0001
UNVA, logMAR	0.033 ± 0.043	0.575 ± 0.215	0.0001
CSS, frequency 3 cpd, units	7.41 ± 0.49	5.66 ± 0.54	0.0001
CSS, frequency 6 cpd, units	7.54 ± 0.45	6.95 ± 0.50	0.01
CSS, frequency 12 cpd, units	7.41 ± 0.45	6.83 ± 0.37	0.01
CSS, frequency 18 cpd, units	7.41 ± 0.45	6.02 ± 0.55	0.0001
Threshold of retina sensitivity, dB	27.9 ± 1.1	24.6 ± 3.5	0.01
Implicit time VEP on flash, msec	32.1 ± 14.5	29.1 ± 10.5	0.19
Amplitude VEP on flash, μV	99.7 ± 6.4	100.6 ± 8.2	0.17
Implicit time a-wave max ERG, msec	15.7 ± 7.5	21.3 ± 11.0	0.001
Amplitude a-wave max ERG, μV	149.7 ± 36.0	139.8 ± 42.7	0.17
Implicit time b-wave max ERG, msec	36.2 ± 3.2	45.9 ± 6.6	0.0001
Amplitude b-wave max ERG, μV	311.8 ± 57.7	287.0 ± 63.4	0.027
FFT, Hz	34.7 ± 2.1	32.3 ± 2.9	0.001

*UDVA* = uncorrected distance visual acuity; *UNVA* = uncorrected near visual acuity; *CSS* = contrast spatial sensitivity; *VEP* = visual evoked potentials; *ERG* = electroretinogram; *FFT* = flicker fusion threshold

A significant decrease of the contrast sensitivity in low and high spatial frequency ranges, the average values of the b-wave ERG maximum amplitude, the flicker fusion threshold and an increase in the a-wave and b-wave ERG maximum latency were found in patients with presbyopia.

### Binocularity

The change in the perception Lang stereo tests was not significant.

A study of binocular cooperation showed an inhibition of the near physiological diplopia in 20% of the patients with presbyopia.

There were multiple changes. These include a significant distortion of the binocular interaction zone with the reduction of total binocularity area, shift in the space towards the near focal point, and the fusion neutralization in the convergence zone. The changes in the proximal, distal and convergence fusion borders were determined (Table 3).

**Table 3:** Comparisons of Fusion Reflex Characteristics in Study Groups (M ± SD)

Characteristics	Control	Presbyopia	*P*-value
Proximal fusion border, cm	5.23 ± 1.58	18.90 ± 6.85	0.0001
Distal fusion border, cm	90.05 ± 7.35	69.20 ± 12.21	0.0001
Convergence fusion border, 10^−1^ cm	24.83 ± 6.14	30.31 ± 6.91	0.0001
Divergence fusion border, 10^−1^ cm	63.38 ± 3.55	58.21 ± 5.61	0.056
Length of binocularity field, cm	84.78 ± 7.75	50.82 ± 16.05	0.0001
Width of binocularity field, 10^−1^ cm	41.88 ± 3.35	30.39 ± 6.34	0.0001
Area of binocularity field, cm^2^	365.60 ± 45.10	174.40 ± 87.70	0.0001

**Fig. 4:**
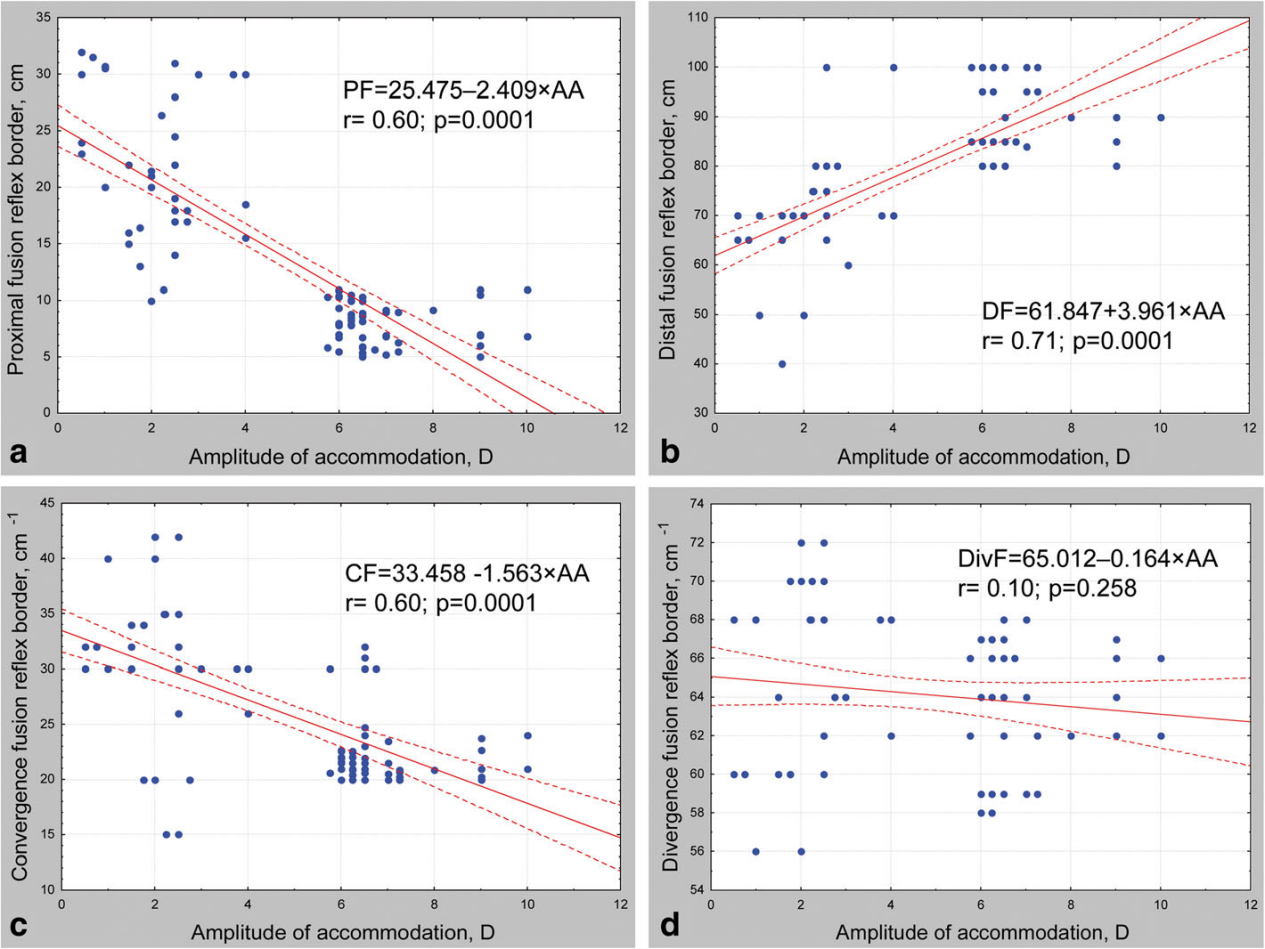
Regressions between Fusion Reflex Borders and Accommodation Amplitude. **a** relationship between proximal fusion border and accommodation amplitude, **b** relationship between distal fusion border and accommodation amplitude, **c** relationship between convergence fusion border and accommodation amplitude, **d** relationship between divergence fusion border and accommodation amplitude

**Fig. 5:**
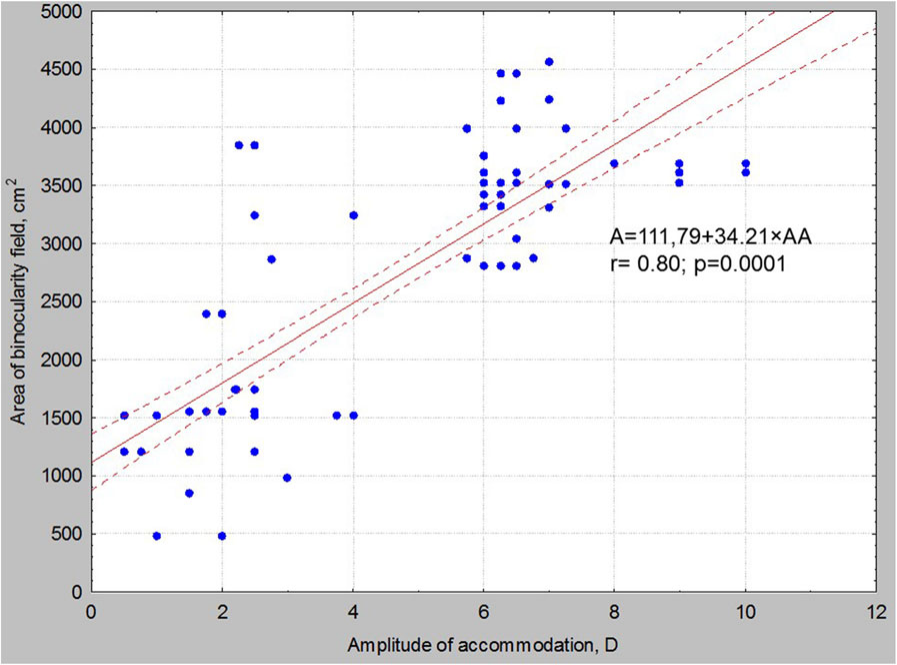
Regressions between Binocularity Field Area and Accommodation Amplitude

The regressions between disparate fusion reflex limits and accommodation amplitude are shown in Fig. 4. In the first (control) group, the mean area of binocularity field was 365.6 ± 45.1 cm^2^ (minimum 280 cm^2^, maximum 456 cm^2^). All patients with presbyopia had a decreased fusion ability with the mean area of binocularity field at 174.4 ± 87.7 cm^2^ (minimum 48 cm^2^, maximum 385 cm^2^). The regression between binocularity field area and accommodation amplitude is shown in Fig. 5.

**Fig. 6:**
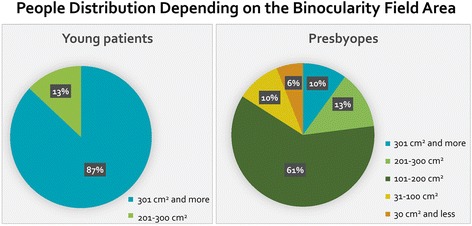
Distribution of Patients Depending on the value of Binocularity Field Area

It is interesting to note that 77% patients with presbyopia had binocular suppression in some grade, while 6% of patients had extreme decrease in the binocularity field area (Fig. 6).

### Intrasystem interactions

The multivariate kinds of statistical analyses were used for the understanding of the vision reception transformation in presbyopia formation.

**Fig. 7:**
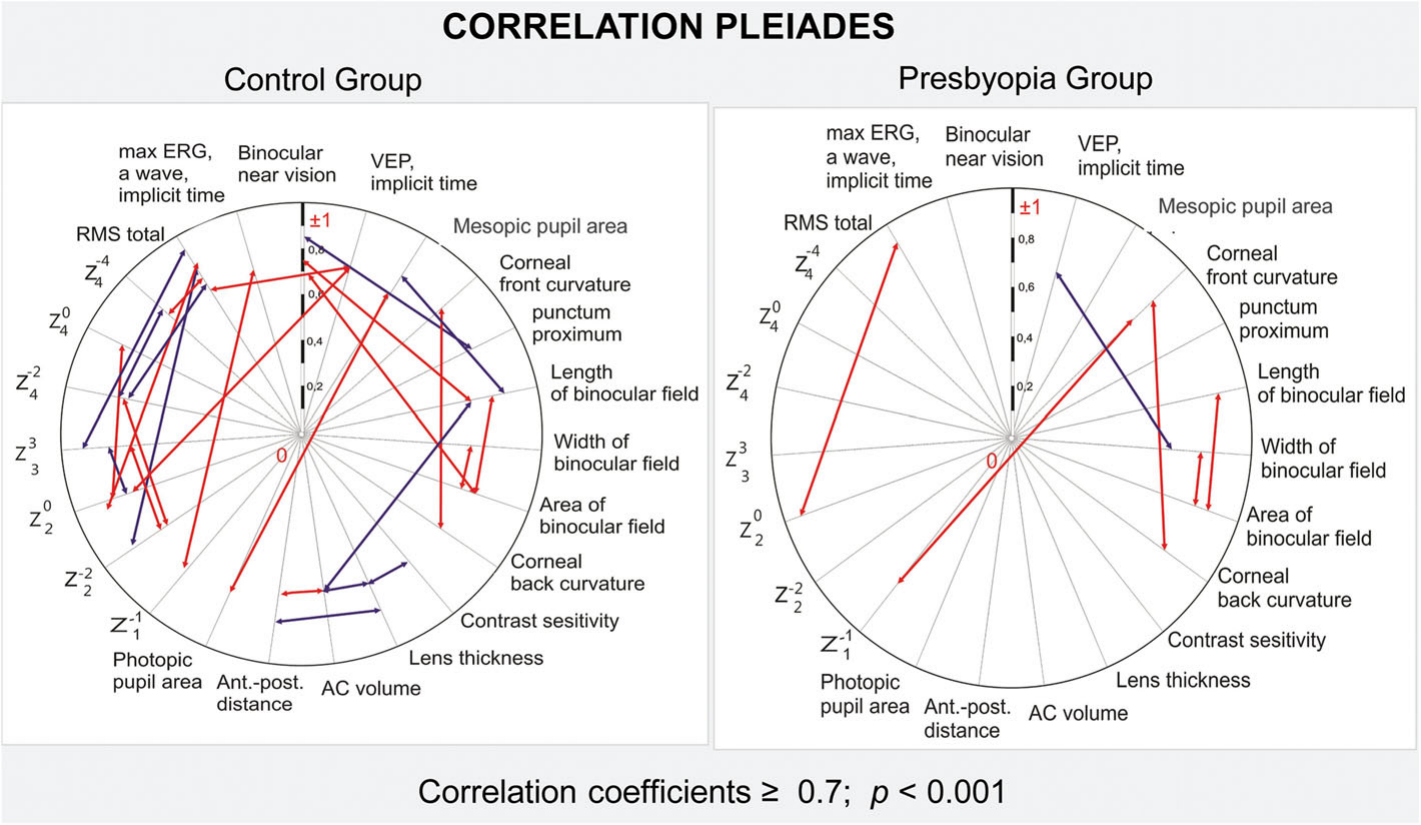
Correlation Pleiades of Control and Presbyopia Groups

A Pearson correlation analysis of visual system parameters was made. The comparison of the correlation Pleiades (correlations with *P*-value equal or less than 0.001) within Control and Presbyopia Groups revealed a reduction in the strength of most relationships. The correlation Pleiades are represented in Fig. 7, where the positive correlations were shown as red arrows and the negative correlations as blue ones.

It is evident that young people without presbyopia have a much larger number of interdependencies between structural and functional indicators of the visual system than in patients with presbyopia. In young people, 25 close relationships were established, but in patients with presbyopia, there were only 6 such relationships. Instead of destroyed relationships, there was a new correlation between the width of the binocularity field and the implicit time of visual evoked potentials (*r* = −0,7; *p* = 0.001).

In the forward stepwise discriminant analysis, eight indices were selected for 100% discrimination of studied groups. The matrix of most informative variables for discrimination is represented in Table 4.

**Table 4:** Matrix of most informative variables for discrimination studied groups

Discriminant Function Analysis Summary Step 8, Wilks’ Lambda:0.02771, approx. F (9.74) = 288.46 Mahalanobis D^2^ = 139.84, *p* < 0,0001				
Variables	Wilks’ Lambda	F-remove (1.74)	*p*-level	Tolerance
UNVA	0.068917	110.0151	0.000001	0.621721
Proximal border of fusion field	0.049343	57.7521	0.000001	0.667574
Amplitude of accommodation	0.040528	34.2152	0.000001	0.619955
Binocularity area	0.038421	28.5871	0.000001	0.567583
Coefficient corneal spherical aberration Z_4_^0^	0.037609	26.4206	0.000002	0.545072
Pupil diameter in photopic conditions	0.033739	16.0866	0.000143	0.718888
Implicit time b-wave max ERG, msec	0.030783	8.1942	0.005462	0.803496
CSS, frequency 3 cpd, units	0.029672	5.2271	0.025101	0.850332

The separation between groups is not only due to the accommodation state but also to some other significant changes in the structural and functional parameters. The indicators of the tolerance show that all features are orthogonal and their contributions to the separation do not overlap.

The results of the discriminant analysis showed that the fusional ability made the high contribution in the separation of two groups. At the same time, the contribution of other sensory parameters in the division was less expressed.

### Discussion

This research aimed to describe the transformation of the visual system functional organization during the presbyopia formation. The results of the study broaden our understanding of the presbyopia mechanisms. It was found that the structural and functional state of the visual system in middle-aged patients with presbyopia is significantly different from young people.

**Fig. 8:**
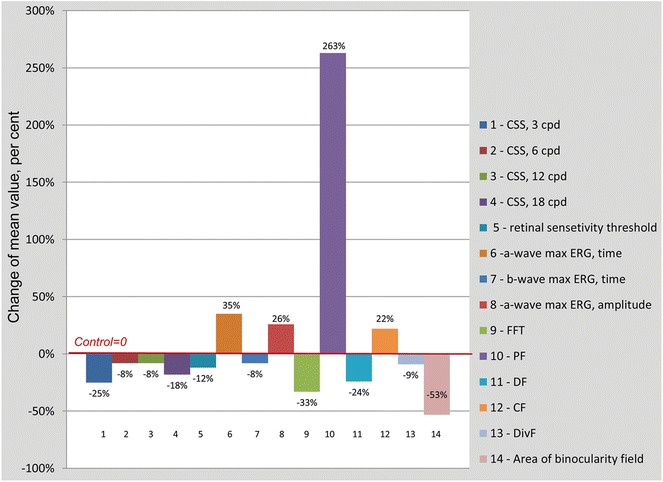
The Changes of the visual parameters (M) in patients with presbyopia in comparison with indicators of young people

The reduction of the accommodation is significant, but not the only sign of visual transformation in patients with presbyopia. The increase in the number of optical errors despite the pupil tour decrease worsens the conditions for the formation of the retinal image. The formation of presbyopia is accompanied by the misalignment of visual sensory processing with varying degrees of functional defect severity (Fig. 8).

The decrease of the contrast sensitivity at frequencies below four cpd reflects the amplification of the visual system internal noise (at the level of receptive fields). Whereas the contrast sensitivity at high spatial frequencies is limited with optical parameters (aberrations, diffraction phenomenon or “sampling noise” of the visual image). The patients with presbyopia had the signs both internal noise and sampling noise (noise of signal processing). The change of temporal parameters of the visual system at middle age patients indicates the initial deficiency of interactions between neurons and the signs of the central nervous system fatigue. These changes can be viewed as signs of aging.

The new data about binocular visual system activity was established. The processes of binocular interaction inhibition make a significant contribution to the misalignment of the visual perception. The area of binocularity field, where the disparate fusion is possible, was reduced twice. The variation of proximal fusion limit and a decrease of the binocularity field area are more serious than the variation in other sensor parameters.

On the one hand, this phenomenon may be a result of age-related changes in the neurons. On the other hand, the inhibition process can be motivated by the desire to liberate the body from the excessive flow of the visual information under the shortage of accommodation. The process of efferent synthesis is an active selection of information aimed to release biologically significant flows and is formed in such integral efferent excitations that are required by the body in a given situation.

Our results correspond to the relevant studies. Granger-Donetti revealed that majority of presbyopes had disorders of binocular cooperation in some degree due to a decrease in the slow convergence [42]. The accommodation amplitude decrease is accompanied by the increase of vergence movement latency, the reduction of the vergence fusion and the speed of the fast vergence [43].

In this study, we analyzed data in people with the visual system that mostly meets the ideal. Even in this situation, 77% of presbyopia patients had binocularity shortage. In 6% of cases, there were profound signs of the deep inhibition processes. These data are likely useful in presbyopia surgery. The analysis of the surgery results using monovision or multifocal optical strategies in patients with an extreme deficit of binocularity is the next step of research.

## Conclusions

The presbyopia formation is accompanied by a significant reorganization of the visual system activity and the creation of the new visual processing interactions. It was revealed that 77% of the presbyopia patients had binocularity shortage. The overall reduction of binocularity field area in extreme grade was seen in 6% of cases. These data may have implications for presbyopia surgery.
